# Spontaneous Riboflavin-Overproducing *Limosilactobacillus reuteri* for Biofortification of Fermented Foods

**DOI:** 10.3389/fnut.2022.916607

**Published:** 2022-06-09

**Authors:** Irina Spacova, Sarah Ahannach, Annelies Breynaert, Isabel Erreygers, Stijn Wittouck, Peter A. Bron, Wannes Van Beeck, Tom Eilers, Abbas Alloul, Naïm Blansaer, Siegfried E. Vlaeminck, Nina Hermans, Sarah Lebeer

**Affiliations:** ^1^Research Group Environmental Ecology and Applied Microbiology (ENdEMIC), Department of Bioscience Engineering, University of Antwerp, Antwerp, Belgium; ^2^Natural Products and Food Research and Analysis (NatuRA), Department of Pharmaceutical Sciences, University of Antwerp, Antwerp, Belgium; ^3^Research Group of Sustainable Energy, Air and Water Technology, Department of Bioscience Engineering, University of Antwerp, Antwerp, Belgium

**Keywords:** *Lactobacillus*, lactobacilli, vitamin B2, vitamin-producing bacteria, fermentation, dairy alternative, plant drink, vegan

## Abstract

Riboflavin-producing lactic acid bacteria represent a promising and cost-effective strategy for food biofortification, but production levels are typically insufficient to support daily human requirements. In this study, we describe the novel human isolate *Limosilactobacillus reuteri* AMBV339 as a strong food biofortification candidate. This strain shows a high natural riboflavin (vitamin B2) overproduction of 18.36 μg/ml, biomass production up to 6 × 10^10^ colony-forming units/ml (in the typical range of model lactobacilli), and pH-lowering capacities to a pH as low as 4.03 in common plant-based (coconut, soy, and oat) and cow milk beverages when cultured up to 72 h at 37°C. These properties were especially pronounced in coconut beverage and butter milk fermentations, and were sustained in co-culture with the model starter *Streptococcus thermophilus*. Furthermore, *L. reuteri* AMBV339 grown in laboratory media or in a coconut beverage survived in gastric juice and in a simulated gastrointestinal dialysis model with colon phase (GIDM-colon system) inoculated with fecal material from a healthy volunteer. Passive transport of *L. reuteri* AMBV339-produced riboflavin occurred in the small intestinal and colon stage of the GIDM system, and active transport *via* intestinal epithelial Caco-2 monolayers was also demonstrated. *L. reuteri* AMBV339 did not cause fecal microbiome perturbations in the GIDM-colon system and inhibited enteric bacterial pathogens *in vitro*. Taken together, our data suggests that *L. reuteri* AMBV339 represents a promising candidate to provide riboflavin fortification of plant-based and dairy foods, and has a high application potential in the human gastrointestinal tract.

## Introduction

Riboflavin (vitamin B2; 7,8-dimethyl-10-ribityl-isoalloxazine) is a water-soluble vitamin with several key functionalities required for adequate energy levels, healthy reproductive function, lactation, and successful pregnancy outcomes in humans [EFSA Panel on Dietetic Products and Nutrition and Allergies EFSA NDA Panel, (1)]. Because the human body cannot produce riboflavin, this vitamin or its active forms, flavin mononucleotide (FMN) and flavin adenine dinucleotide (FAD), must be ingested regularly. Riboflavin is found predominantly in products of animal origin such as milk, eggs, and meat, and in lower amounts in green leafy vegetables (2). The daily average requirements as set by the European Food Safety Authority (EFSA) is 1.3 mg/day for adults, 1.5 mg/day for pregnant women, 0.5 mg/day for children aged 1–3 years, 1.4 mg/day for children aged 15–17 years, and an additional 0.31 mg/day requirement for lactating women (3). Riboflavin deficiency is a global concern, as it is prevalent both in developing (e.g., Côte d’Ivoire, Kenya, Zambia) (4–6) as well as developed (e.g., Canada, United Kingdom) countries (7, 8), especially in populations with a lower intake of foods of animal origin (2).

Fortification with riboflavin increases food nutritional value and uptake of micronutrients, which can help prevent riboflavin deficiencies (9, 10). Riboflavin can be obtained from natural or genetically modified overproducer microorganisms, including filamentous fungi and yeasts such as *Eremothecium ashbyii*, *Eremothecium gossypii*, and *Candida famata*, as well as bacteria such as *Bacillus subtilis* and genetically modified *Escherichia coli* (11, 12). However, due to safety concerns and/or suboptimal organoleptic properties, the use of these microorganisms is limited to industrial-scale riboflavin production, followed by purification prior to riboflavin addition to food products (13). Consequently, alternative safe and riboflavin-overproducing microbial strains that can be directly used for natural biofortification of a wide range of foods are highly sought-after (11, 14).

Beneficial lactobacilli are a promising alternative to fortify foods with vitamins. Riboflavin production is a highly strain-specific property of lactobacilli (14). While some probiotic strains such as *Lacticaseibacillus rhamnosus* GG (14, 15) cannot produce this vitamin, riboflavin production levels up to 3–5 μg/ml have been described for natural riboflavin-producing lactobacilli isolates such as *Lactiplantibacillus plantarum* strains M5MA1-B2 and HY7715 grown under standard non-optimized laboratory conditions (16–18). The ability of specific lactobacilli to increase riboflavin content in plant-based alternatives for milk is especially important, since these alternatives gain worldwide popularity, yet they often lack specific vitamins. A recent USDA Branded Food Products Database (BFPDB) showed that only 16% of plant-based alternatives for milk contained riboflavin (19). Lactic acid bacteria also significantly add to the flavor and texture profiles of the fermentation end-products resulting from animal milk and plant-based substrates. Moreover, the acidification capacity of these bacteria can inhibit pathogenic growth, typically resulting in longer shelf life (20). In addition to the ability of the lactobacilli to produce riboflavin in a range of food matrices, it can be beneficial if the selected strains survive the gastrointestinal tract, so that the vitamins can be produced *in situ* at the target site to further enhance delivery and maximize the chance of reaching required daily intake levels (16, 21).

In this study, we investigate the unique riboflavin-overproducing properties of *Limosilactobacillus reuteri* AMBV339 isolated from a healthy volunteer and explore its application potential for biofortification of fermented beverages, with or without the model starter *Streptococcus thermophilus*. An important goal was to explore whether *L. reuteri* AMB339 could be used to produce riboflavin in plant-based beverages for which riboflavin fortification is especially relevant (19). In addition to riboflavin overproduction, the strain’s gastrointestinal survival and interactions with the human fecal microbiome in a simulated gastrointestinal dialysis model are evaluated.

## Materials and Methods

### Bacterial Strains and Culturing Conditions

Bacterial strains used in this study included human vaginal isolates *Limosilactobacillus reuteri* AMBV336, *L. reuteri* AMBV339, and *L. reuteri* AMBV371 from healthy Isala project participants (22). Strains of lactobacilli from commercially available probiotic products included *L. reuteri* RC-14 (23), *Lacticaseibacillus rhamnosus* GG (15), *Lacticaseibacillus paracasei* Immunitas, *L. paracasei* Shirota, *Lactiplantibacillus pentosus* KCA1 (24), *L. rhamnosus* GR-1, *L. plantarum* WCFS1 (25), and *Lactobacillus helveticus* 1807. Commercially available strains from bacterial culture collections such as the American Tissue Culture Collection (ATCC) and Belgian Coordinated Collections of Microorganisms (BCCM) included *Lactobacillus sakei* ATCC15521, *Lacticaseibacillus zeae* DSM20178, *L. paracasei* JCM8130, *L. rhamnosus* LC705, *Lactobacillus acidophilus* LMG8151, *Lactiplantibacillus mudanjiangensis* DSM28402, *Latilactobacillus graminis* LMG9825, and *Latilactobacillus curvatus* LMG9198. The following strains of lactobacilli have previously been isolated from fermented foods (26): *L. reuteri* AMBF471, *Ligilactobacillus salivarius* AMBF472, *Lactiplantibacillus pentosus* AMBF473, *Limosilactobacillus fermentum* AMBF478, and *L. fermentum* AMBF479. Commercial yogurt isolate *Streptococcus thermophilus* LMG18311 was obtained *via* BCCM. *S. thermophilus* AMBF701 was also previously isolated from a commercial yogurt (27). Pathogens *Listeria monocytogenes* LMG23194 and *Shigella sonnei* LMG10473 were available *via* BCCM, while *Salmonella enterica* subsp. *enterica* serovar Typhimurium ATCC14028 was obtained *via* ATCC.

Strains of lactobacilli were cultured in de Man, Rogosa and Sharpe (MRS) medium at 37°C, 5% CO_2_, in static conditions. *Listeria monocytogenes* LMG 23194, *Shigella sonnei* LMG10473, and *Salmonella enterica* subsp. *enterica* serovar Typhimurium ATCC 14028 were cultured in brain heart infusion (BHI) medium at 37°C in shaking conditions (284 rpm). To separate cell-free culture supernatant from cell pellets, the overnight cultures of lactobacilli in MRS broth were centrifuged at 15.000 × g for 10 min at 4°C, and the supernatant was filter sterilized (0.20 μm cellulose acetate membrane filter, VWR).

### Genome Sequencing and Bioinformatics Analysis

The method for genome isolation of *L. reuteri* was based on the P3 protocol in Alimolaei and Golchin (28). Whole-genome sequencing (WGS) was performed using the Nextera XT DNA Sample Preparation kit (Illumina) and the Illumina MiSeq platform, using 2 × 250 cycles, at the Laboratory of Medical Microbiology (University of Antwerp, Antwerp, Belgium) (29). Genome assembly was performed with SPAdes version 1.0.4.^1^ Genome completeness was checked using checkM v1.0.11 (30). The Average Nucleotide Identity (ANI) between bacterial genomes was assessed using the orthoANI (Orthologous Average Nucleotide Identity) tool of EzBioCloud (31). The *L. reuteri* RC-14 genome was downloaded *via* refseq (GCF_002762415.1). The *L. reuteri* AMBV339 genome was uploaded and annotated with the Pathosystems Resource Integration Center (PATRIC) genome annotation tool^2^ (32, 33) and key genes for riboflavin metabolism were identified *via* the Kyoto Encyclopedia of Genes and Genomes database (KEGG) PATHWAY database.^3^ Screening of the genome sequence against FPbase (The Fluorescent Protein Database)^4^ was performed (34). The genome was scanned for antimicrobial resistance (AMR) genes with abricate v1.0.1^5^ using the Resfinder database.

### Fluorescence Spectrum Analysis and Assessment of Riboflavin Production by Lactobacilli

Lactobacilli were cultured in corresponding media (MRS broth or food matrices), and (as indicated in the results) either the whole culture, the cell-free culture supernatant, or the cell pellet washed in phosphate-buffered saline (PBS) and resuspended in MRS broth were analyzed. To obtain a whole spectrum, measurements were performed in 96-well plates on 200 μl of whole culture per well with the Synergy H1 multimode reader with a fixed excitation of 350 nm, and emission start at 380 nm and emission stop at 700 nm in steps of 10 nm. To assess riboflavin concentrations based on fluorescence, sample measurements were performed in 96-well plates on 200 μl/well with the Synergy HTX multimode reader. Excitation was set at 485 nm and emission was measured at 528 nm. In all experiments, a sterile medium control was included.

### High-Performance Liquid Chromatography-Ultraviolet Analysis

Samples were analyzed using an HPLC-system from Agilent (1260 Quaternary pump and auto-injector, 1,290 temperature controller, 1,200 UV-detector) connected with an eclipse plus C18 column (4.6 × 100 nm, 3.5 μm). A mobile phase consisting of MeOH (26.5% v/v)-Na_2_HPO_4_.12H_2_O (10g/l, 73.5% v/v), pH 3.5 with H_3_PO_4_, was used isocratically at a flow of 1 ml/min. The injection volume was 80 μl and run time was 15 min. The UV detector was set at 260 nm and retention time of riboflavin standard was 8.3 min. Riboflavin (Sigma-Aldrich PHR-1054-1G, lot LRAC0268; CAS 83-88-5) was used for preparing the standard. The standard solutions for calibration ranged from 0.041 to 2.615 μg/ml (dilutions were made in milliQ, stock standard riboflavin was dissolved in 12% perchloric acid). Dialysate samples were ready for analysis. Other samples were subjected to an extraction procedure with perchloric acid (12%). After adding perchloric acid (1/2 dilution), samples were vortexed (1 min) and put in darkness for 15 min before centrifugation (732 × g, 10 min, 23°C). A solution of 2M K_2_CO_3_ in KOH (6M) was added to the supernatant at 4.28% (v/v), vortexed and centrifuged (1800 × g, 10 min, 23°C) before HPLC-analysis.

### Gas Chromatography Analysis

Ten clean Erlenmeyer flasks containing 300 ml of MRS broth and a magnetic stir bar were closed by an open topped screw cap with silicone rubber seal (DWK Life Sciences; PTFE-protected) before autoclaving. *L. reuteri* AMBV339, *L. reuteri* AMBV336, and *L. reuteri* RC-14 were inoculated in 10 ml of MRS broth, in triplicate, and incubated overnight at 37°C with 5% CO_2_, together with sterile MRS broth as a control. After vortexing, three replicate overnight cultures were combined in a 50 ml falcon. Optical density (OD) measurement at 600 nm was performed. Six ml of each combined culture was centrifuged in triplicate for 10 min at 621 × g at 4°C, washed once in PBS (1x) and the pellet was resuspended in 5 ml MRS broth from the corresponding Erlenmeyer flask. Each resuspended pellet was added to the corresponding Erlenmeyer flask followed by tightly closing. All Erlenmeyer flasks were placed on a magnetic stirrer, followed by the first sampling of liquid (1 ml of triplicates and 10 ml of sterile MRS) and gas (2 × 4 ml of sterile MRS), and incubated at 37°C without CO_2_. Sampling of liquid (1 ml) and gas (2 × 4 ml) was performed after 2, 4, 6, and 24 h, using a 22G × 11/2′′, 0.7 mm × 40 mm needle (BD MicroLance™ 3) and a Luer Lock Syringe of 10 ml (SOL-MTM). In addition, pressure measurements were performed after 6 and 24 h to decide on pressure relief.

Gas samples were analyzed using gas chromatography (GC-2014) to monitor the gas composition, all separately in single runs on a Shincarbon-ST 50/80 column with a thermal conductivity detector (Shimadzu, Japan) operating at 200°C and 50 mA. Argon was used as carrier gas at 35 ml/min. Samples of approximately 3 ml were injected at 200°C which suffices to completely fill the sample loop, with excess being rejected by the system. Prior to every new single run, the injection port was flushed with the carrier gas. The column temperature was set at 100°C for CO_2_ detection (approximately 6 min/run). To monitor the presence of H_2_, N_2_, and O_2_, a column temperature of 40°C was used (approximately 4 min/run).

### Bacterial Strain Culturing in Food Matrices

Single bacterial strains or their combinations were inoculated in commercial food matrices widely available in Belgian supermarkets and cultured at 37°C for 72 h, with sampling at 24, 48, and 72 h. Plant-based alternatives included coconut (Suzi Wan^®^ coconut “milk”), soy (Alpro Soya Original drink) and oat (Alpro “Not MLK” oat-based drink) beverages. Animal milk-based beverages included whole cow milk (Boni Selection), semi-skimmed cow milk (Boni Selection), and butter milk (Balade). Nutritional composition of the different food matrices is depicted in Table 1. Fluorescence-based assessment of riboflavin levels at different time points (e.g., 24, 48, 72 h) was performed on 200 μl/well of culture using the Synergy HTX multimode reader cfr. “Fluorescence spectrum analysis and assessment of riboflavin production by lactobacilli.” Measurements of pH were performed at the final time point using the Mettler Toledo SevenCompact pH meter. When applicable, determination of colony-forming units (CFU)/ml was performed by plating out serial dilutions of cultures in sterile PBS on MRS agar.

**TABLE 1:** Nutritional composition of food matrices used in this study.

	Coconut beverage	Soy beverage	Oat beverage	Cow milk (whole)	Cow milk (semi-skimmed)	Butter milk
Energy (kJ)	790	163	247	273	199	152
Energy (kcal)	191	39	59	65	47	36
Fat (g)	19	1.8	3.5	3.6	1.6	0.4
Saturated fatty acids (FA) (g)	18	0.3	0.4	2.3	1	0.2
Monounsaturated FA (g)	Not listed	0.4	Not listed	Not listed	Not listed	Not listed
Polyunsaturated FA (g)	Not listed	1.1	Not listed	Not listed	Not listed	Not listed
Carbohydrates (g)	3.1	2.5	5.7	4.8	4.8	4.8
Sugars (g)	2.4	2.5	0	4.8	4.8	4.8
Fibers (g)	<0.5	0.5	1	0	0	0.4
Proteins (g)	2.2	3	0.7	3.4	3.4	3
Salt (g)	0.04	0.09	0.12	0.13	0.11	0.2
**Vitamins**						
Vit D (μg)	Not listed	0.75	0.75	Not listed	Not listed	Not listed
Vit B2 (mg)	Not listed	0.21	Not listed	Not listed	Not listed	Not listed
Vit B12 (μg)	Not listed	0.38	0.38	Not listed	Not listed	Not listed
**Minerals**						
Calcium (mg)	Not listed	120	120	120	120	Not listed

### Survival of *Galleria mellonella* Larvae Injected With Bacteria

*Galleria mellonella* larvae were purchased from Anaconda reptiles (Kontich, Belgium), the larvae were then stored at 4°C and were used within 7 days. Safety testing was performed as described in De Boeck et al. (35), with 15 larvae used per treatment group. The safety of bacteria was tested by injecting 10 μl of a 10^3^ CFU/ml bacterial solution in the last prolegs of the larvae and monitoring larvae survival for 144 h. PBS was used as a negative control. Baseline viability of the larvae was 100% as checked in a group without injection.

### Riboflavin Transport Assessment in Caco-2 Epithelial Cell Monolayers

Testing was performed according to the protocol described in Mortelé et al. (36). Briefly, Caco-2 cells were maintained in Dulbecco’s Modified Eagle Medium (DMEM, Gibco) with 10% foetal calf serum (FCS), non-essential amino acids and PenStrep at 37°C, 5% CO_2_. Cells were seeded on ThinCerts at approximately 0.6 × 10^6^ viable cells/ml 21–29 days before the experiment. On the day of the experiment, either an *L. reuteri* suspension at 10^7^ CFU/ml DMEM without supplements or riboflavin at 1 mg/ml was added to the apical compartment of the ThinCerts together with 10% of respective cell-free culture supernatant containing bacteria-derived riboflavin. Riboflavin transport through Caco-2 was recorded by measuring the riboflavin fluorescence in 100 μl of DMEM medium from the basolateral compartment at regular time points for 24 h cfr. “Fluorescence spectrum analysis and assessment of riboflavin production by lactobacilli” described above.

### Gastric Juice Survival of *Limosilactobacillus reuteri* AMBV339

*L. reuteri* AMBV339 preculture in MRS was inoculated in 300 ml of MRS broth divided over 6 × 50 ml tubes and incubated overnight at 37°C with 5% CO_2_. The culture was centrifuged for 10 min at 7425 × g at 4°C. Subsequently, the supernatant was discarded and the pellet was washed with 2 ml PBS and resuspended in 34 ml PBS. After the addition of 16.5 ml pepsin (1.34 g), the pH of all suspensions was adjusted with HCl (6 M) to pH values of 2, 3, and 4, respectively, all in duplicate. Subsequently, the suspensions entered the stomach stage of the optimized Gastrointestinal Dialysis Model (GIDM-colon). Longitudinal sampling was performed at six time points of 5, 10, 30, 60, and 90 min. Samples were plated out on MRS agar in a ten-fold dilution series to determine CFU/ml.

### Gastrointestinal Dialysis Model With Colon Phase

A previously developed and validated gastrointestinal dialysis model with colon phase (GIDM-colon) was used to mimic human digestion and biotransformation processes (37). For each experiment, the model was inoculated with feces provided by a healthy donor and stored frozen. This study involving human participants was reviewed and approved by the Committee of Medical Ethics UZA/UAntwerpen, Belgium (approved on the 8th of July 2019, number EC UZA 19/26/310). The participants provided their written informed consent to participate in this study.

*L. reuteri* AMBV339 culture was pretreated as aforementioned and administered as bacteria resuspended in saline (0.9% NaCl solution), or as a culture in coconut beverage fermented by *L. reuteri* AMBV339 for 72 h. Briefly, during simulation of the gastric stage *L. reuteri* AMBV339 resuspended in saline, or as a culture in coconut beverage (34 ml, *n* = 3), was subjected to pepsin solution (2052.6 U/ml digest), pH 3, at 35–37°C, stomach motion and digestion time of 90 min. Coconut beverage or saline without *L. reuteri* AMBV339 was included as control sample. The small intestinal and colon stage were performed using Amicon stirred ultrafiltration cells (model 8,200, 200 ml, 63.5 mm diameter; dialysis cells), equipped with a dialysis membrane (ultrafiltration discs, Ultracel MWCO 1000 Da, 63.5 mm diameter) to allow passive diffusion mimicking the one-way absorption from lumen through mucosa.

The content of the gastric stage was manually transferred to ultrafiltration cells to simulate the small intestinal stage and 50 ml of ultrapure water was added. Dialysis bags (molecular weight cut-off of 12–14 kDa; Visking size 6 Inf Dia 27/32–21.5 mm) containing 1 M NaHCO_3_ were used to gradually alter the pH from 3 to 7.5 in 30 min. Ultrafiltration cells were placed in a water bath (35–37°C), continuously stirred and connected with a water tank and a N_2_ gas input using push bottom control switches. N_2_ gas puts pressure (2 bar) on the ultrafiltration cells to enable dialysis. After 30 min of dialysis, 15 ml of a porcine pancreatin-bile solution [0.4% (w/v) of pancreatin (32,000 FIP-U lipase, 143 600 FIP-U amylase, 16 400 FIP-U protease), and 0.766% (w/v) of bile in 0.1 M NaHCO_3_] was added to each ultrafiltration cell. Small intestinal digestion lasted 90 min.

In order to simulate the ascending colon stage, the pH was adjusted to 5.8 using 1 M HCl and ultrafiltration cells were transferred to an anaerobic glove box (0.5% O_2_, 35–37°C). 50 ml of a 10% (v/v) human fecal slurry suspension was added to each ultrafiltration cell with exception of the negative control. Instead, 50 ml of sterile phosphate buffer solution was added to the negative control. Ultrafiltration cells were continuously stirred and pressure was introduced on top of the ultrafiltration cells (0.8 bar N_2_) to obtain dialysis. Samples for microbiome analysis (2 ml) were taken after 0, 2, 4, 6, 24, 48, and 72 h, and dialysis was performed during 2, 4, 6 h of colon stage. Samples for CFU and riboflavin levels determination were taken during all GIDM stages. Each sample was divided in a total of three aliquots: (i) for HPLC analysis, centrifuged for 8 min at 18,226 × g and the supernatant was stored on -20°C prior to analysis; (ii) for 16S analysis, transported in eNAT™ buffer (Copan, Brescia, Italy), intended for microbiome profiling, and stored on -20°C prior to analysis; (iii) for immediate cultivation of microorganisms.

### Microbiome Analysis in a Gastrointestinal Dialysis Model With Colon Phase *via* 16S rRNA Amplicon Sequencing

Samples from the colon stage of the GIDM-colon system for microbiome analyses, taken after 0, 2, 4, 6, 24, 48, and 72 h, were thawed before further processing. These samples were then thoroughly vortexed and DNA was extracted with the DNeasy PowerSoil Pro Kit (Qiagen, Hilden, Germany) according to the instructions of the manufacturer. DNA concentration of all samples was measured using the Qubit 3.0 Fluorometer (Life Technologies, Ledeberg, Belgium) according to the instructions of the manufacturer. No less than 2 μl of each bacterial DNA sample was used to amplify the V4 region of the 16S rRNA gene, using standard barcoded forward (515F) and reverse (806R) primers. These primers were altered for dual index paired-end sequencing, as described in Kozich et al. (38). The resulting PCR products were checked on a 1% agarose gel. The PCR products were then purified using the Agencourt AMPure XP Magnetic BeadCapture Kit (Beckman Coulter, Suarlee, Belgium) and the concentration of all samples was measured using the Qubit 3.0 Fluorometer. Next, a library was prepared by pooling all PCR samples in equimolar concentrations. This library was loaded onto a 0.8% agarose gel and purified using the NucleoSpin Gel and PCR clean-up (Macherey-Nagel). The final concentration of the library was measured with the Qubit 3.0 Fluorometer. Afterwards, the library was denatured with 0.2N NaOH (Illumina, San Diego California United States), diluted to 6 pM and spiked with 10% PhiX control DNA (Illumina). Finally, dual-index paired-end sequencing was performed on a MiSeq Desktop sequencer (Illumina).

### Inhibition of Enteric Pathogen Growth by *Limosilactobacillus reuteri* Supernatants

Testing was performed cfr. (35). Briefly, pathogen cultures of *Listeria monocytogenes* LMG 23194, *Shigella sonnei* LMG10473, and *Salmonella enterica* subsp. enterica serovar Typhimurium ATCC 14028 were diluted 1:100 in BHI medium in a microtiter plate and cell-free supernatants of *L. reuteri* strains grown overnight in MRS broth were added at 1:5 dilution. Ampicillin was used as control at 100 μg/ml. Growth of pathogens in triplicate wells was longitudinally measured with the Synergy HTX multimode reader at OD of 600 nm, and area under the growth curve (AUC) was calculated.

### Bioinformatics Analysis of Microbiome Data

Quality control and processing of reads was performed using the R package DADA2, version 1.8.0. Briefly, this entailed quality filtering of the reads (allowing maximum two expected sequencing errors per read pair), dereplication, denoising, merging of forward and reverse reads (read pairs with base conflicts were removed), removal of chimeras and read classification. The merged and denoised reads (amplicon sequence variants or ASVs) were taxonomically annotated from the phylum to the genus level with the assignTaxonomy function of DADA2 using the EzBioCloud reference 16S rRNA database (39). All data handling and visualization was performed in R version 3.4.4 (40) using the tidyverse set of packages and the in-house package tidyamplicons^6^ (41).

### Statistical Analysis

All data except microbiome data were analyzed in GraphPad Prism version 9.2.0. Statistical analysis on data with several variables was performed by two-way ANOVA with Tukey’s multiple comparisons test. In other cases, one-way ANOVA with Dunnett’s multiple comparisons test was used. Statistical analysis of larvae survival was performed using the Log-rank (Mantel-Cox) test.

### Data Availability Statement

Bacterial genome and microbiome data is available in the European Nucleotide Archive (ENA) under accession number PRJEB52196, and GCA_940926095 for the *L. reuteri* AMBV339 genome assembly. The R code generated during this study can be found on GitHub^7^.

## Results

### Fluorescent *Limosilactobacillus reuteri* AMBV339 Harbors the Genetic Capacity for Riboflavin Production

In the large-scale Isala citizen science project^8^ (22), we isolated a bacterial strain with a remarkable yellow-green fluorescent phenotype from a healthy female volunteer. The fluorescence phenotype of this strain appeared to be unique among the approximately 2,000 vaginal isolates obtained so far. The strain’s yellow-green fluorescent phenotype was most apparent when exposed to UV light after overnight growth in MRS broth and on MRS agar. The strain was subsequently subjected to whole-genome sequencing and designated *L. reuteri* AMBV339. Analysis of the *L. reuteri* AMBV339 genome revealed a genome size of 1.99 Mbp and a G + C content of 38.7%, with 99.46% completeness. Another *L. reuteri* strain that did not exhibit a clear fluorescent phenotype upon visual inspection was isolated from the same woman and designated *L. reuteri* AMBV336. Based on the calculated Average Nucleotide Identity (ANI) between their genomes, *L. reuteri* AMBV339 was more closely related to *L. reuteri* AMBV336 (ANI > 99.99%) compared to the model probiotic strain *L. reuteri* RC-14 (ANI = 96.06%).

The PATRIC annotation tool was implemented to annotate the genome of *L. reuteri* AMBV339, and the annotated gene list was screened for compounds with putative yellow-green fluorescence properties (42). Considering that flavins are known to emit yellow-green fluorescence (42), and since fluorescence detection in the yellow-green spectrum (emission ∼520 nm) is a standard method to screen bacterial isolates for riboflavin production (43), a flavin-overproducing phenotype was suspected. Genome analysis with the KEGG (Kyoto Encyclopedia of Genes and Genomes) database indicated that *L. reuteri* AMBV339 indeed harbors all genes required for riboflavin production, which was confirmed in the gene list annotated in PATRIC (Table 2). The possibility of fluorescent proteins such as the green fluorescent protein being produced by *L. reuteri* AMBV339 was excluded, as no significant hits for fluorescent molecules in the yellow-green spectrum were found in the annotated gene list or *via* the FPbase (The Fluorescent Protein Database).

**TABLE 2:** Riboflavin biosynthesis pathway genes present in the *L. reuteri* AMBV339 genome.

Gene product involved in riboflavin synthesis or metabolism	Gene ontology (GO) in PATRIC	Contig	Start	End	Length, bp
**Genes required for riboflavin synthesis according to the KEGG database**					
3,4-dihydroxy-2-butanone 4-phosphate synthase (EC 4.1.99.12)/GTP cyclohydrolase II (EC 3.5.4.25)	GO:0008686/ GO:0003935	1598.1216.con.0004	70,789	71,970	1,182
Diaminohydroxyphosphoribosylaminopyrimidine deaminase (EC 3.5.4.26)/5-amino-6-(5-phosphoribosylamino) uracil reductase (EC 1.1.1.193)	GO:0008835/ GO:0008703	1598.1216.con.0004	69,136	70,194	1,059
Hydrolase, HAD superfamily (3.1.3.104)		1598.1216.con.0013	834	1,652	819
6,7-dimethyl-8-ribityllumazine synthase (EC 2.5.1.78)		1598.1216.con.0004	71,973	72,431	459
Riboflavin synthase eubacterial/eukaryotic (EC 2.5.1.9)	GO:0004746	1598.1216.con.0004	70,187	70,789	603
FMN adenylyltransferase (EC 2.7.7.2)/Riboflavin kinase (EC 2.7.1.26)	GO:0003919/ GO:0008531	1598.1216.con.0016	22,505	23,452	948
**Additional genes involved in riboflavin transport or metabolism**					
RibT protein, riboflavin biosynthesis acetyltransferase (GNAT) family		1598.1216.con.0004	10,251	10,607	357
Substrate-specific component RibU of riboflavin ECF transporter		1598.1216.con.0004	13,069	13,647	579

### *Limosilactobacillus reuteri* AMBV339 Displays a Riboflavin Overproduction Phenotype

**FIGURE 1:**
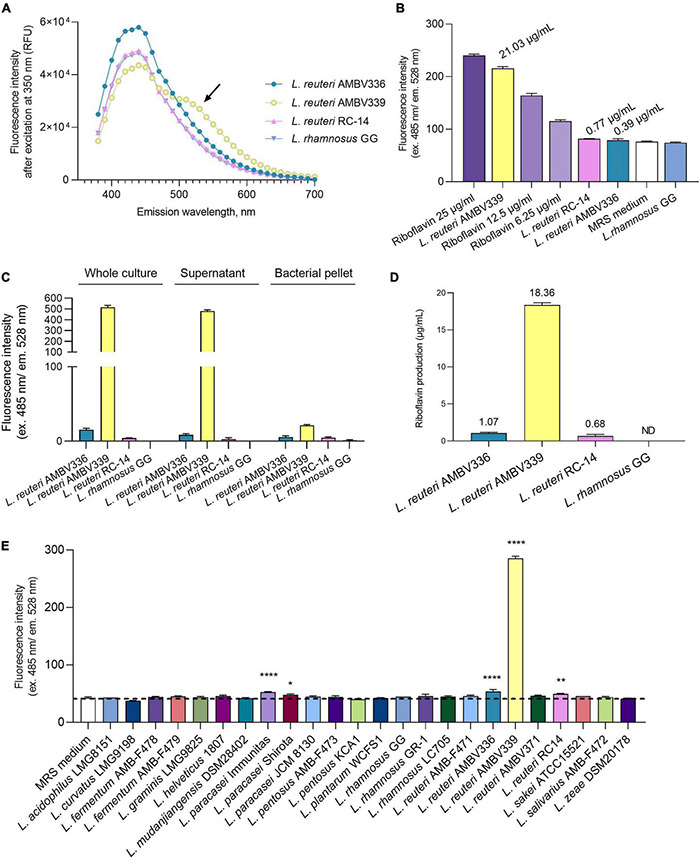
Riboflavin production by *L. reuteri* AMBV339 in MRS medium compared to other lactobacilli. **(A)** Emission spectra of *L. reuteri* AMBV336, *L. reuteri* AMBV339, *L. reuteri* RC-14 and *L. rhamnosus* GG cultures. Arrow points to an additional emission peak in the *L. reuteri* AMBV339 spectrum between 500 and 550 nm. Data is presented as means; RFU: relative fluorescence units. **(B)** Fluorescence characteristic of riboflavin in MRS cultures of *L. reuteri* AMBV336, *L. reuteri* AMBV339, *L. reuteri* RC-14, and *L. rhamnosus* GG compared to riboflavin control. Data is presented as means ± SD, riboflavin controls are 25, 12.5, and 6.25 μg/mL MRS medium. Approximate levels of riboflavin determined *via* linear regression are indicated above the bars. **(C)** Fluorescence characteristic of riboflavin in whole culture, cell-free culture supernatant and bacterial pellet of *L. reuteri* AMBV336, *L. reuteri* AMBV339, *L. reuteri* RC-14, and *L. rhamnosus* GG. Data is normalized to the MRS medium background and presented as means ± SD. **(D)** Riboflavin concentration in culture supernatants of *L. reuteri* AMBV336, *L. reuteri* AMBV339, *L. reuteri* RC-14, and *L. rhamnosus* GG determined by HPLC-UV. Data is presented as means ± SD; ND: not detected. **(E)** Fluorescence characteristic of riboflavin quantified in cultures of various probiotic and other lactobacilli. Data is presented as means ± SD. *p < 0.05, **p < 0.01, ****p < 0.0001 as determined by One-way ANOVA with Dunnett’s multiple comparisons test compared to the MRS medium control (depicted by the dotted line).

To experimentally confirm whether riboflavin or related compounds were overproduced by *L. reuteri* AMV339, we determined the fluorescence emission spectrum from 350 to 700 nm for the *L. reuteri* AMBV339 culture in MRS medium. This emission spectrum was selected to cover a range of several potential fluorescent molecules, including nicotinamide adenine dinucleotide (NADH), tryptophan and flavins (42). The emission spectrum of *L. reuteri* AMBV339 was compared to the spectra of three non-fluorescent strains: *L. reuteri* AMBV336 isolated from the same woman (this study), commercial probiotic *L. reuteri* RC-14 (23), and the model probiotic *L. rhamnosus* GG, of which the latter is known to lack the capacity for riboflavin production (14, 15). The fluorescence emission spectrum of the *L. reuteri* AMBV339 culture showed a unique peak between 500 and 550 nm (Figure 1A), which was in line with the suspected emission peak of riboflavin at approximately 520 nm (42, 43).

Therefore, we subsequently used more specific filters to ensure excitation at 485 nm and confirmed the high emission of the *L. reuteri* AMBV339 culture at 528 nm (Figure 1B). The fluorescence intensity obtained for *L. reuteri* AMBV339 was close to the riboflavin control at 25 μg/ml, while *L. reuteri* AMBV336, *L. reuteri* RC-14, and *L. rhamnosus* GG culture fluorescence in MRS was much less pronounced. Using simple linear regression on the fluorescence data after subtracting the baseline MRS fluorescence, the average approximate concentration of the fluorescent compound was determined to be 21.03 ± 0.53 μg/ml in *L. reuteri* AMBV339 cultures, 0.77 ± 0.11 μg/ml in *L. reuteri* RC-14 cultures, 0.39 ± 0.43 μg/ml in *L. reuteri* AMBV336 cultures, and 0 μg/ml in *L. rhamnosus* GG cultures.

The fluorescence measurements were subsequently used to localize the riboflavin-like production of *L. reuteri* AMBV339 (Figure 1C). The fluorescence was mostly concentrated in the *L. reuteri* AMBV339 culture supernatant and not in the cell pellet, suggesting secretion of the fluorescent compound by this strain. To validate that this fluorescence was due to riboflavin production, more specific high-performance liquid chromatography-ultraviolet (HPLC-UV) analysis with pure riboflavin (C_17_H_20_N_4_O_6_; CAS 83-88-5) as reference was conducted on the cell-free culture supernatants, detecting riboflavin at 18.36 ± 0.31 μg/ml in *L. reuteri* AMBV339 cultures grown overnight in MRS medium (Figure 1D). Cultures of *L. reuteri* AMBV336 contained 1.07 ± 0.12 μg riboflavin/ml, cultures of *L. reuteri* RC-14 contained 0.68 ± 0.22 μg riboflavin/ml and production of riboflavin by *L. rhamnosus* GG in MRS medium was undetectable with the HPLC-UV method used. These concentrations approximately correspond with the concentrations determined *via* fluorescence (Figure 1B).

As we showcased fluorescence (ex. 485 nm and em. 528 nm) as a quantitative indicator for riboflavin production, we employed fluorescence measurements to compare the riboflavin production capacity of *L. reuteri* AMBV339 in MRS with a range of commercially available probiotic and other lactobacilli strains, such as isolates from fermented foods (“AMBF” strains) from our strain biobank (*n* = 23). The *L. reuteri* AMBV339 culture fluorescence reflecting riboflavin production was by far the highest compared to all other tested lactobacilli (Figure 1E).

### *Limosilactobacillus reuteri* AMBV339 Produces Riboflavin in Specific Plant-Based and Milk Beverages

As riboflavin fortification is especially relevant for plant-based beverages, we also aimed to assess whether *L. reuteri* AMBV339 could be implemented in combination with the model starter *Streptococcus thermophilus*, which is used for conventional yogurt production to improve the fermentation process and organoleptic properties of the final product, and it is also increasingly implemented for plant-based fermented food production (44). Thus, riboflavin production based on fluorescence, and pH-lowering properties reflecting fermentation by *L. reuteri* AMBV339, *S. thermophilus* LMG18311 or their combination, were measured longitudinally (24–72 h) in plant-based beverages (coconut, soy, and oat beverages) and milk products (buttermilk, whole, and semi-skimmed milk).

**FIGURE 2:**
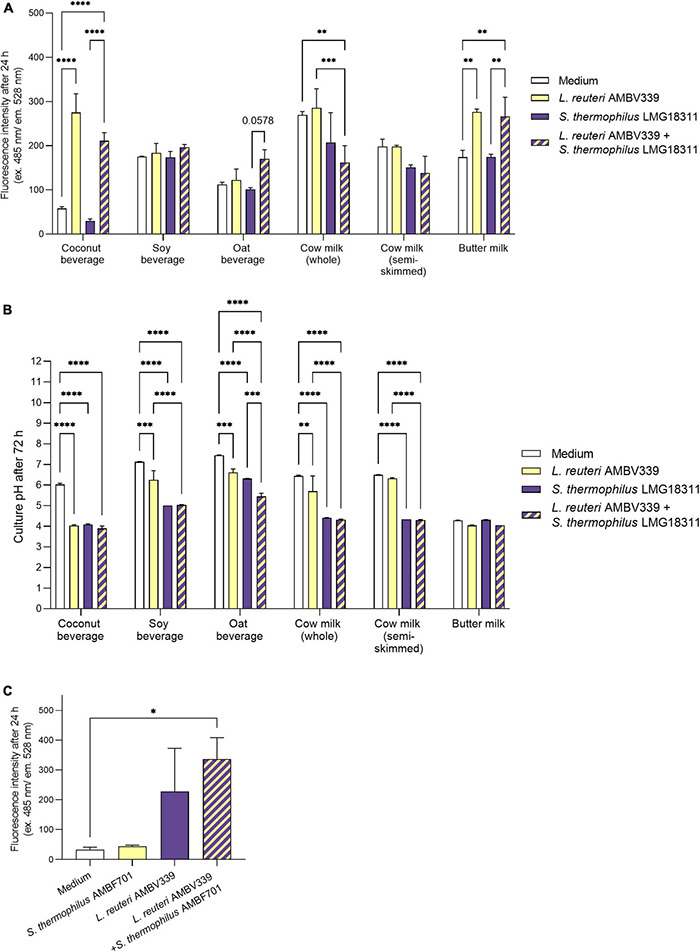
**(A)** Riboflavin production in 24 h cultures of *L. reuteri* AMBV339 and *S. thermophilus* LMG18311 alone or combined in coconut, soy and oat beverages, whole and semi-skimmed cow milk, and butter milk, and **(B)** final culture pH after 72 h. **(C)** Riboflavin production in 24 h cultures of *L. reuteri* AMBV339 and *S. thermophilus* AMBF701 alone or co-cultured in a coconut beverage. Data is presented as means ± SD. *p < 0.05, ^**^p < 0.01, ^***^p < 0.001, ^****^p < 0.0001 or depicted *p*-value, as determined by two-way ANOVA with Tukey’s multiple comparisons test. Comparisons were made with the medium (food matrix as such) condition, or with the *L. reuteri* AMBV339 + *S. thermophilus* conditions.

Based on fluorescence measurements, *L. reuteri* AMBV339 alone significantly increased the riboflavin content of the coconut beverage (4.7-fold) and butter milk (1.6-fold) after 24 h (Figure 2A), as well as after 48 and 72 h (Supplementary Figures 1A,B) of culturing compared to medium baseline. In the soy beverage (which was already fortified with 2.1 mg/l riboflavin), a 1.4-fold increase in riboflavin fluorescence was only observed in the culture of *L. reuteri* AMBV339 after 48 h (Supplementary Figure 1A). The largest increase in riboflavin due to *L. reuteri* AMBV339 relative to medium baseline was observed in the coconut beverage, while the highest absolute riboflavin fluorescence was obtained for *L. reuteri* AMBV339 cultured in butter milk.

The combination of *L. reuteri* AMBV339 and *S. thermophilus* LMG18311 resulted in a marked increase in riboflavin fluorescence as compared to the medium baseline in buttermilk (1.5-fold), coconut (3.6-fold) and oat (1.5-fold) beverages after 24 h of incubation (Figure 2A). Similar results were obtained after 48 and 72 h of incubation (Supplementary Figures 1A,B). Thus, in the oat beverage, the combination of *L. reuteri* AMBV339 with *S. thermophilus* LMG18311 yielded even higher riboflavin fluorescence increase compared to *L. reuteri* AMBV339 alone. Of note, *S. thermophilus* LMG18311 itself did not increase the riboflavin content of any of the tested beverages. In soy beverage and cow milk, it seemed to consume riboflavin after 24–72 h (Figure 2 and Supplementary Figures 1A,B). Riboflavin production by the combination of *L. reuteri* AMBV339 and *S. thermophilus* LMG18311 was not observed in the tested cow milk (whole and semi-skimmed) and in the tested soy beverage, all three of which already had high baseline riboflavin concentrations among the tested beverages.

In parallel, the pH-lowering properties of *L. reuteri* AMBV339 reflecting fermentation of the selected food matrices were assessed, which is important for the organoleptic and antimicrobial properties of the final product (44). pH also affects riboflavin stability and its fluorescence-based detection, since maximum riboflavin fluorescence is observed in pH ranges of 4–8 (45). Lowering of the final pH, indicating fermentation by *L. reuteri* AMBV339 (Figure 2B), was significant in coconut (final pH = 4.03 ± 0.05), soy (final pH = 6.26 ± 0.44), and oat beverages (final pH = 6.62 ± 0.16), and in whole cow milk (final pH = 5.71 ± 0.74), but not in semi-skimmed cow milk (final pH = 6.33 ± 0.03). The already low pH of butter milk remained virtually identical after fermentation by *L. reuteri* AMBV339 (pH = 4.05 ± 0.03). *S. thermophilus* LMG18311 alone was more efficient than *L. reuteri* AMBV339 in the acidification of soy and oat beverages, and whole and semi-skimmed cow milk. Compared to *L. reuteri* AMBV339, the combination of *L. reuteri* AMBV339 and *S. thermophilus* LMG18311 resulted in significantly lower final culture pH in soy (final pH = 5.04 ± 0.01) and oat (final pH = 5.45 ± 0.16) beverages, whole cow milk (final pH = 4.32 ± 0.03), and semi-skimmed cow milk (final pH = 4.30 ± 0.02) (Figure 2B).

To validate the promising data on riboflavin production in coconut beverage, an additional strain of *S. thermophilus* AMBF701 previously isolated from commercial yogurt (27) was also tested. Similar observations regarding riboflavin production were made in coconut beverage: the combination of *S. thermophilus* AMBF701 with *L. reuteri* AMBV339 even resulted in a trend toward higher riboflavin production compared to *L. reuteri* AMBV339 alone, suggesting a synergy between *L. reuteri* AMBV339 and certain *S. thermophilus* strains that leads to higher riboflavin levels (Figure 2C).

### Riboflavin Overproduction by *Limosilactobacillus reuteri* AMBV339 Does Not Affect Biomass Generation and Fermentation in Beverages With the Highest Riboflavin Production

In addition to riboflavin production and pH-lowering capacity reflecting fermentation, the capacity of bacteria to survive and generate sufficient biomass is an important industrial parameter for fermented food production. Conventional fermented dairy products such as yogurts contain live lactic acid bacteria at approximately 10^7^–10^8^ CFU/ml and higher (44). Thus, we next evaluated the capacity of *L. reuteri* AMBV339 to generate biomass in the coconut beverage and butter milk, the food matrices in which the highest riboflavin production was observed. To specifically assess whether the riboflavin overproduction phenotype affected the biomass generation and pH-lowering capacity of *L. reuteri* AMBV339, two other strains capable of only weak riboflavin production were included for comparison: the closely related *L. reuteri* AMBV336 and the commercial probiotic *L. reuteri* RC-14. MRS broth was also included as the optimal laboratory medium for *L. reuteri*. The data was aligned with final fluorescence measurements reflecting riboflavin production, and final pH measurements reflecting fermentation.

**FIGURE 3:**
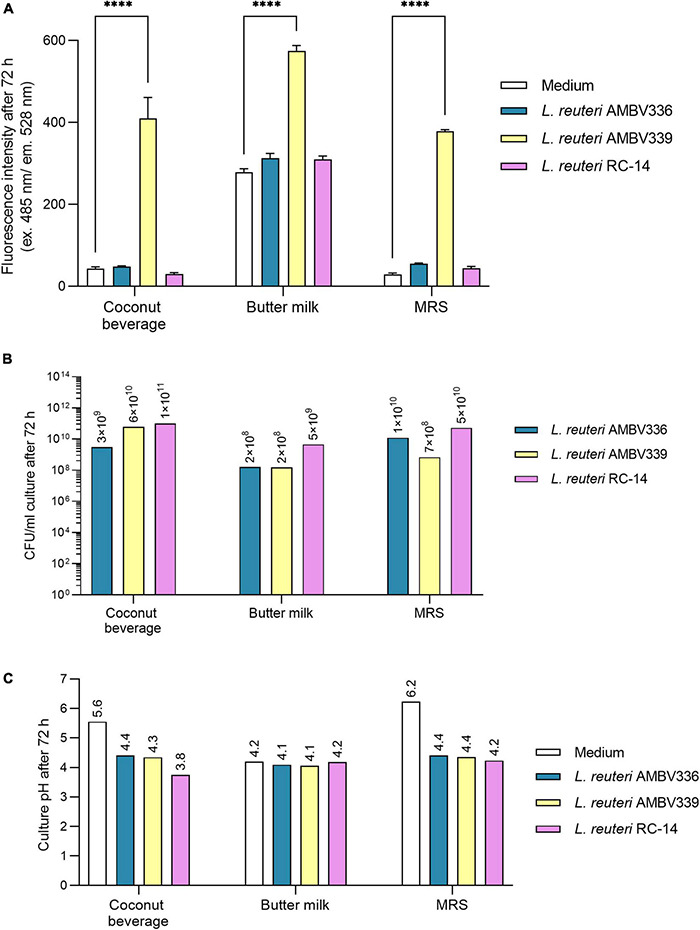
**(A)** Riboflavin production, **(B)** biomass generation, and **(C)** pH lowering capacity reflecting fermentation in cultures of *L. reuteri* AMBV339 compared to *L. reuteri* AMBV336 and *L. reuteri* RC-14 in coconut beverage, butter milk, and MRS broth after 72 h at 37°C. Data is presented as means ± SD. ^****^p < 0.0001, as determined by two-way ANOVA with Tukey’s multiple comparisons test.

*L. reuteri* AMBV339 led to a significant increase in fluorescence reflecting higher riboflavin concentrations in the coconut beverage, butter milk and MRS broth, which was not the case for *L. reuteri* AMBV336 and *L. reuteri* RC-14 (Figure 3A). The fluorescence in cultures of *L. reuteri* AMBV339 in the coconut beverage and butter milk was similar to or higher than in MRS broth, suggesting riboflavin levels of 18.36 μg/ml or above based on the previously obtained HPLC-UV data in MRS cultures.

*L. reuteri* AMBV339 generated biomass ranging from 10^8^ CFU/ml in butter milk to 10^10^ CFU/ml in the coconut beverage, which was generally similar to *L. reuteri* AMBV336 and *L. reuteri* RC-14 (Figure 3B). Also, the final pH of *L. reuteri* AMBV339 cultures in the coconut beverage, butter milk and MRS broth was similar to the pH of *L. reuteri* AMBV336 and *L. reuteri* RC-14 cultures in these matrices (Figure 3C).

In addition to (lactic) acid production reflected in the lowering of pH, carbon dioxide (CO_2_) should also be generated during fermentation by heterofermentative lactobacilli. To assess differences in fermentative metabolism, batch growth experiments with gas chromatography analysis on the headspace of *L. reuteri* AMBV339, *L. reuteri* AMBV336, and *L. reuteri* RC-14 cultures in MRS broth were implemented. *Limosilactobacillus* species are heterofermentative, and anaerobic or aerotolerant (46). During cultivation, almost no oxygen was consumed in the cultures of *L. reuteri* AMBV339, *L. reuteri* AMBV336, and *L. reuteri* RC-14 (Supplementary Table 1). After 24 h, the CO_2_ content increased from 0 to approximately 63 volume percentage for all three strains, suggesting that anaerobic chemotrophy was the dominant metabolism. The strong CO_2_ production after 24 h was accompanied by a surge in pressure (Supplementary Table 1), while the N_2_ and O_2_ levels remained stable over 24 h suggesting an ongoing fermentation without significant differences between the strains.

### *Limosilactobacillus reuteri* AMBV339 Survives in Simulated Gastric Juice and Gastrointestinal Dialysis Model

Considering the high potency of *L. reuteri* AMBV339 for riboflavin fortification, fermentation, and biomass production in relevant food matrices, the next goal was to explore this strain for potential *in vivo* applications targeting the human gastrointestinal tract (GIT). We validated by an *in silico*-based analysis that the *L. reuteri* AMBV339 genome shows no antibiotic resistance genes using the abricate tool, and no virulence factors according to the Virulence Factor Database (VFDB) (47). We also phenotypically showed the absence of toxicity/virulence using a novel *Galleria melonella* invertebrate larvae model, which is increasingly used to screen pathogen toxicity (48). Larvae injected with *L. reuteri* AMBV339 showed no significant drop in survival over the course of 6 days compared to larvae injected with PBS, in contrast to larvae injected with the pathogen *Listeria monocytogenes* that showed a significant drop in survival starting from the first day (Supplementary Figure 2).

**FIGURE 4:**
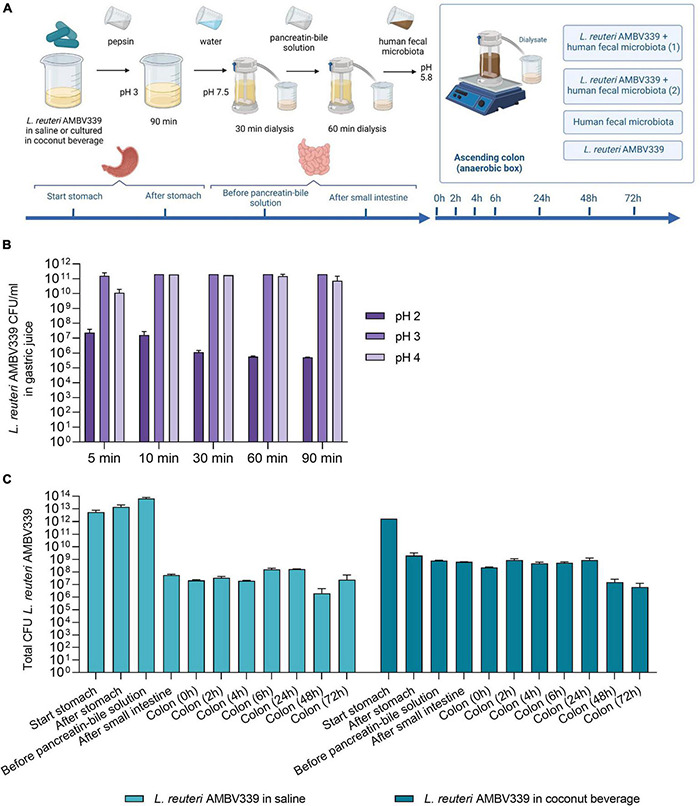
**(A)** Set-up of *L. reuteri* AMBV339 testing in the simulated gastrointestinal dialysis model (GIDM-colon), and longitudinal survival of *L. reuteri* AMBV339 in **(B)** gastric juice at different pH, or **(C)** in different parts of the simulated gastrointestinal tract model inoculated with human fecal microbiota. For the simulated gastrointestinal tract *L. reuteri* AMBV339 in a 0.9% NaCl solution (saline) or *L. reuteri* AMBV339 in fermented coconut beverage was added. Data is presented as means with standard deviation. Data for gastric juice survival at pH 4 at 30 min was extrapolated from adjacent time points.

Considering the envisioned application of *L. reuteri* AMBV339 in the human GIT, the survival of *L. reuteri* AMBV339 was evaluated in a validated simulated gastrointestinal dialysis model with colon stage (GIDM-colon) (37) (Figure 4A). The passage of metabolically active *L. reuteri* AMBV339 into the intestinal tract could facilitate riboflavin production/uptake in the small and large intestine where it can be absorbed (49) and/or benefit the resident microbial communities. *L. reuteri* AMBV339 survived up to 90 min in simulated gastric juice at pH 2, 3, and 4, reflecting different stomach acidity levels before, during and after food intake, respectively (Figure 4B). The highest *L. reuteri* AMBV339 survival rates were at pH 3 and 4 with up to 10^11^ CFU/ml; however, even after 90 min at pH 2 the strain was still viable at 10^6^ CFU/ml.

In addition, *L. reuteri* AMBV339 was evaluated in two experimental set-ups in the GIDM-colon system: (1) *L. reuteri* AMBV339 administered in saline (0.9% NaCl), and (2) *L. reuteri* AMBV339 administered as a culture in fermented coconut beverage. *L. reuteri* AMBV339 survived for at least 72 h when administered in saline or in fermented coconut beverage in the GIDM-colon system (Figure 4C). For example, after addition of 10^12^ CFU of *L. reuteri* AMBV339 culture in coconut beverage to the system (a reasonable amount of lactobacilli in 100 ml of fermented beverage based on our results), 2 × 10^9^CFU were detected after the stomach stage. Furthermore, 6.3 × 10^8^CFU of *L. reuteri* AMBV339 were present after the small intestine stage, and 8.8 × 10^8^CFU and 6.3 × 10^6^CFU were still detected in the colon compartment after 24 and 72 h, respectively.

### Uptake of *Limosilactobacillus reuteri* AMBV339-Derived Riboflavin in a Gastrointestinal Dialysis Model and Intestinal Epithelial Cells

**FIGURE 5:**
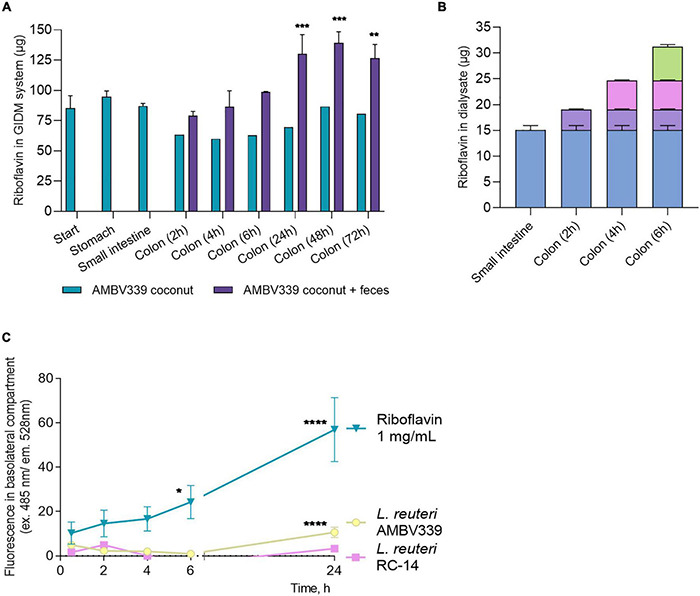
**(A)** Total amount of riboflavin in the different parts of the GIDM-colon system after addition of *L. reuteri* AMBV339 culture in coconut beverage without (“AMBV339 coconut”) or with the human fecal microbiome (“AMBV339 coconut + feces”); Data is presented as means ± SD. **p < 0.01, ***p < 0.001 compared to the riboflavin levels measured at the start of the GIDM system. **(B)** Riboflavin transfer into dialysate by passive diffusion from the small intestine and colon stages of the GIDM-colon system (measured up to 6h in this experimental set-up). **(C)** Active transport of riboflavin produced by *L. reuteri* AMBV339 through intestinal epithelial Caco-2 monolayers. Pure riboflavin as such was used for comparison (riboflavin 1 mg/ml condition). Data is presented as means ± SD per time point. *p < 0.05, **p < 0.01, ***p < 0.001, ****p < 0.0001 compared to the MRS medium control.

To benefit the consumer, the riboflavin generated by *L. reuteri* AMBV339 needs to be taken up by the human GIT, thus production and passive transport of riboflavin produced by *L. reuteri* AMBV339 was evaluated in the GIDM system, while active transport was assessed *via* human intestinal Caco-2 monolayers. First, the total amount of riboflavin in the GIDM-colon system was measured with HPLC-UV at different stages and time points (Figure 5A), comparing two conditions: *L. reuteri* AMBV339 culture in coconut beverage with or without the addition of the human fecal microbiota. *L. reuteri* AMBV339 cultured in coconut beverage and administered to the GIDM-colon system led to an increase in riboflavin concentrations at the colon stage in the presence of fecal microbiota compared to the starting riboflavin concentrations and compared to the condition without the fecal microbiota (Figure 5A). Specifically, a marked increase in the total amount of riboflavin was observed at 24 h of the colon stage of the GIDM. At 48 h of the colon stage the amount of riboflavin in the model increased by approximately 57 μg (± 9 μg) compared to the start of the digestion experiment (Figure 5A). No riboflavin was detected in the fecal control sample not containing *L. reuteri* AMBV339 (data not shown). Also, when *L. reuteri* AMBV339 cells in saline were added to the GIDM-colon system, no riboflavin production was detected with the HPLC-UV in the GIDM-colon in this experimental set-up (data not shown), highlighting the importance of the food matrix.

To assess whether the riboflavin produced by *L. reuteri* AMBV339 in the coconut beverage matrix could also be absorbed by the gastrointestinal system, passive diffusion of riboflavin from the GIDM system was assessed at the small intestinal and colon stage for up to 6 h. A considerable amount of riboflavin passively diffused from the GIDM containing *L. reuteri* AMBV339 in the coconut beverage matrix in the small intestinal phase (15.04 ± 0.92 μg) and colon phase (16.18 μg in total over 6 h) (Figure 5B) through the semi-permeable dialysis membrane.

Because the GIDM-colon system does not contain human cells, it was only possible to investigate passive riboflavin transport in this system. To additionally validate the possibility of active *L. reuteri* AMBV339-derived riboflavin transport through the human intestinal epithelial cells, which would facilitate riboflavin uptake *in vivo*, we implemented monolayers of the Caco-2 intestinal epithelial cell line grown on ThinCerts. Differentiated Caco-2 cells recapitulate the transporting properties of the small intestinal epithelium key for riboflavin absorption (36, 49). *L. reuteri* RC-14 and riboflavin at 1 mg/ml were used as control conditions. After 24 h of co-incubation of Caco-2 monolayer with live *L. reuteri* AMBV339 in cell medium with 10% bacterial culture supernatant, or riboflavin at 1 mg/ml, riboflavin transport from the apical to the basolateral side of the Caco-2 monolayer was observed, as evidenced by fluorescence increase in the basolateral compartment beneath the Caco-2 monolayer (Figure 5C). No significant riboflavin transport was observed in the *L. reuteri* RC-14 condition. These results suggest that active transport of riboflavin produced by *L. reuteri* AMBV339 could occur through the intestinal epithelial layer.

### *Limosilactobacillus reuteri* AMBV339 Does Not Perturb the Healthy Fecal Microbiome and Inhibits Enteric Pathogens

**FIGURE 6:**
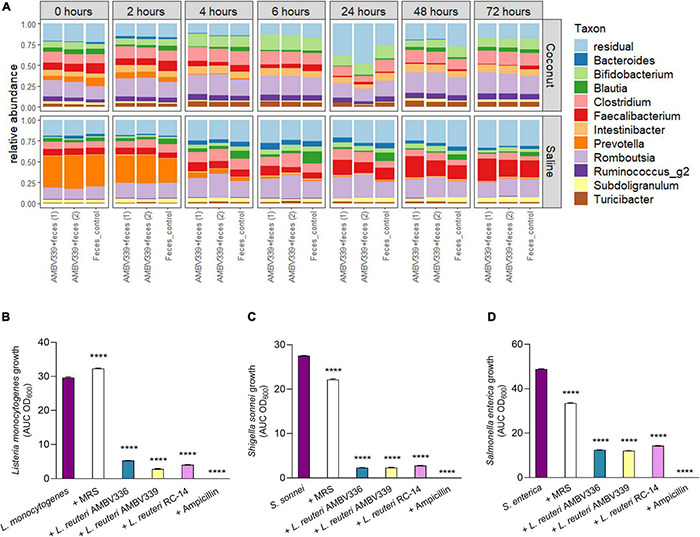
**(A)** Relative taxonomic abundances based on 16S rRNA amplicon sequencing of colon samples to evaluate the influence of *L. reuteri* AMBV339 resuspended in saline or as culture in a fermented coconut beverage on fecal microbiota in the GIDM-colon system. Taxonomic composition of the fecal microbiome on genus-level for *L. reuteri* AMBV339 in saline or coconut culture (in duplicate) and feces control grouped per time point. *L. reuteri* AMBV339 reads were removed, and top 11 genera are visualized, the other taxa are grouped in residual. **(B–D)** Growth inhibition of enteric pathogens **(B)** *Listeria monocytogenes*, **(C)** *Shigella sonnei*, and **(D)** *Salmonella enterica* under influence of supernatants of *L. reuteri* AMBV336, *L. reuteri* AMBV339, and *L. reuteri* RC-14 in MRS. Supernatants were added at 1:5 dilution and MRS medium was used as control. Data is presented as area under the growth curve (AUC) means ± SD per condition based on optical density at 600 nm (OD_600_), ****p < 0.0001 compared to the pathogen control.

After demonstrating that *L. reuteri* AMBV339 could have direct benefits for the host riboflavin levels, we assessed the effects of *L. reuteri* AMBV339 on the human fecal microbiome composition in the GIDM-colon system. Two different experimental conditions were tested in this model, with *L. reuteri* AMBV339 added either as cells resuspended in saline, or as a culture in a fermented coconut beverage (Figure 4A). Longitudinal microbiome assessment was performed by comparing the control fecal microbiome (depicted as “Feces control” in Figure 6A) with the fecal microbiome incubated with *L. reuteri* AMBV339 [this condition was tested in duplicate, depicted as “AMBV339 + feces (1)” and “AMBV339 + feces (2)”]. At 0 h, the average relative abundance of *L. reuteri* was 0% in the feces control condition, while the average relative abundance of *L. reuteri* in the condition where *L. reuteri* AMBV339 was added to the feces was 4.07%. After removing *L. reuteri* AMBV339 reads from the microbiome data (with average relative abundance of 1.25%), the results demonstrated that *L. reuteri* AMBV339 did not shift the taxonomic composition of the fecal microbial communities in the GIDM-colon system (Figure 6A). Further, the microbial community composition within each condition generally did not strongly change over time, besides an increase in residual taxa reads at the 24 h time point in the experiment where *L. reuteri* AMBV339 was added as a culture in a fermented coconut beverage (Figure 6A, top panel). In addition, in both experiments with *L. reuteri* AMBV339 added in either saline or coconut beverage, the detected relative abundance of *Prevotella* was clearly decreased after 2 h in both control fecal microbiome and *L. reuteri* AMBV339 with fecal microbiome. The alpha-diversity, calculated with the Inverse Simpson index (Supplementary Figure 3A), and beta-diversity, calculated as Bray-Curtis dissimilarities on relative abundances (Supplementary Figure 3B), also did not show a strong impact of *L. reuteri* AMBV339 when evaluating the fecal microbiome composition of both control and test conditions over time. When focusing on six dominant genera in the gut samples (i.e., *Bacteroides, Bifidobacterium, Blautia, Clostridium, Faecalibacterium, and Romboutsia*), only minimal differences in relative abundances were present over time and they were comparable for both control and test conditions (Supplementary Figure 3C).

In this study set-up, feces from a healthy participant were inoculated into the GIDM-colon system, therefore it was not possible to test the influence of *L. reuteri* AMBV339 on enteric pathogens. To ensure that riboflavin overproduction did not compromise the potential antimicrobial activity of *L. reuteri* AMBV339 and did not promote enteric pathogen growth, we tested the ability of *L. reuteri* AMBV339 cell-free culture supernatants to inhibit model enteric pathogens *Listeria monocytogenes* LMG 23194, *Salmonella enterica* subsp. *enterica* serovar Typhimurium ATCC 14028, and *Shigella sonnei* LMG10473. The supernatants of *L. reuteri* AMBV336 and *L. reuteri* RC-14 were included for comparison. Supernatants of all three *L. reuteri* strains inhibited all three enteric pathogens (Figures 6B–D and Supplementary Figure 4). *L. reuteri* AMBV339 showed superior inhibition of *Listeria monocytogenes* compared to *L. reuteri* AMBV336 and *L. reuteri* RC-14 (Figure 6B). Detailed growth curves of the pathogens under the influence of lactobacilli supernatants over the course of 21 h are depicted in Supplementary Figure 4. Even though the supernatants of *L. reuteri* AMBV339 contained higher levels of riboflavin compared to *L. reuteri* AMBV336 and *L. reuteri* RC-14, this did not promote *in vitro* enteric pathogen growth at any of the 21 h time points.

## Discussion

In this study, we characterized the healthy human isolate *L. reuteri* AMBV339 with natural riboflavin production levels of 18.36 μg/ml in standard laboratory conditions. Based on the available literature, *L. reuteri* AMBV339 demonstrates the highest riboflavin production levels described for non-modified lactobacilli isolates in standard non-optimized growth conditions (MRS broth, 37°C, 24 h), which so far appear to be in the maximum range of 3–5 μg/ml (14, 16–18). Although genetic modification of the riboflavin biosynthesis pathway in *Lactococcus lactis* NZ9000 resulted in overproduction up to 24 μg/ml of riboflavin (50), live genetically modified strains are currently not allowed to be used for food fortification in the European Union according to the opinion of the EFSA Panel on Genetically Modified Organisms (51). To our knowledge, this is the first study describing an *L. reuteri* strain with a spontaneous riboflavin-overproducing phenotype. Besides the well-reported investigation and applications of overproducing *L. plantarum* strains (16, 18, 52–56) only few other species of food-grade bacteria including *L. lactis* (50), *Leuconostoc mesenteroides, Propionibacterium freudenreichii* (43); *Limosilactobacillus fermentum* (57); *Weissella cibaria*, and *Leuconostoc* spp. (58) have been reported for the vitamin B2 bio-enrichment of fermented foods. Thus, the human isolate *L. reuteri* AMBV339 is a highly promising non-genetically modified strain for efficient riboflavin overproduction among lactobacilli.

Real-life implementation of riboflavin-producing lactobacilli for food biofortification requires that they can efficiently produce this vitamin in relevant food matrices. We have demonstrated that *L. reuteri* AMBV339 significantly increased the riboflavin content in butter milk and a coconut beverage to levels comparable with riboflavin production in MRS broth or higher. The approximate riboflavin levels we obtained with *L. reuteri* AMBV339 in the tested food matrices, especially in coconut beverage, are superior to those previously described for food riboflavin fortification with lactobacilli that yielded riboflavin concentrations in the range of 0.5–6.57 μg/ml (59). According to our results, a 100-ml glass of coconut beverage or butter milk fermented by *L. reuteri* AMBV339 would cover the daily riboflavin needs of children, adolescents and adults set at 0.5, 1.4, and 1.3 mg/day, respectively (3). In our study, riboflavin levels were shown to be sustained or even increased in cultures of *L. reuteri* AMBV339 in food matrices from 24 to 72 h, suggesting a potential shelf-life stability of at least 3 days in the tested conditions. Furthermore, we observed *L. reuteri* AMBV339 growth resulting in 10^8^–10^10^ CFU/ml in the tested food matrices, which is in the typical growth range for model lactobacilli (60, 61). Evidently, its industrial production can be further optimized for higher biomass yields. The biomass production and medium acidification reflecting fermentation by *L. reuteri* AMBV339 was comparable to the non-overproducers of riboflavin *L. reuteri* AMBV336 and *L. reuteri* RC-14, suggesting that the riboflavin overproduction does not lead to a metabolic burden that would negatively impair growth and fermentation properties of *L. reuteri* AMBV339.

We also demonstrated that *L. reuteri* AMBV339 is compatible with the most widely used starter culture species *Streptococcus thermophilus* in a wide range of plant-based and milk beverages, while maintaining its riboflavin-overproducing capacity. Results of co-culturing *L. reuteri* AMBV339 with *S. thermophilus* were in line with the expectations for conventional cow milk-based yogurt products obtained *via* fermentation by *S. thermophilus* and *Lactobacillus delbrueckii* subsp. *bulgaricus.* These products generally have a pH equal to or below 4.5 (important for tartness, thickening and preservation) and a final bacterial density above 10^8^ CFU/ml (62). Our results have a broad implementation potential, since plant-based beverages as alternatives to dairy are growing in popularity and the demand for naturally fortified foods increases (63). Yet, many plant-based beverages such as the standard fermented coconut beverage do not contain detectable riboflavin levels, for example according to the NUBEL Belgian Foods composition table (64). Fermented plant beverages with *L. reuteri* AMBV339 containing high levels of riboflavin could thus address this issue and benefit the health of the general population by increasing riboflavin intake (10, 65).

Besides riboflavin production and fermentation capacity, it is important to consider the gastrointestinal adaptation properties of lactobacilli to efficiently implement them for real-life food biofortification or as beneficial supplements (16, 21). Here, we showed that *L. reuteri* AMBV339 survives in simulated gastric juice, suggesting that it is resistant to the harsh conditions of the stomach before, during and after a meal, and can be transported live to the small intestine where riboflavin is absorbed, as we demonstrated *via* passive transport in the gastrointestinal dialysis model (GIDM-colon) and active transport *via* human intestinal epithelial cells. Importantly, in the GIDM-colon, we observed increased riboflavin production 24 h after *L. reuteri* AMBV339 was added as a culture in coconut beverage, suggesting the possibility of riboflavin production by *L. reuteri* in the context of the human gut. Further testing in humans should be performed to assess whether administration of *L. reuteri* AMBV339 in a specific food matrix such as coconut beverage could stimulate riboflavin production in the gastrointestinal tract.

While riboflavin production and transport were demonstrated after *L. reuteri* AMBV339 was added to the GIDM-colon, no significant disturbances were observed in the healthy fecal donor microbiome. Microbiome analysis within the colon stage of the GIDM suggested that the fecal microbiome composition was influenced by time rather than the addition of *L. reuteri* AMBV339. While the fecal microbial composition did not change, further metabolomic and transcriptomic analyses are encouraged to investigate whether the microbial metabolism was affected by the increased production capacity of riboflavin in the colon after addition of *L. reuteri* AMBV339, such as shown by Hong et al. for *L. plantarum* HY7715 (66). However, gut microbiome modulation is not necessarily a prerequisite for beneficial effects of administered lactobacilli and their metabolic products (67), especially not in healthy donors. Of note, our analysis found no antibiotic resistance genes on mobile elements within the genome of *L. reuteri* AMBV339, however, an assessment of bacterial susceptibility to antimicrobials according to the EFSA guidelines is advised before large-scale implementation of this strain in food and/or feed to exclude the potential for antimicrobial resistance transfer to other microbiome members (68). It is also of interest to further study the effects of *L. reuteri* AMBV339 on more pathogenic fecal donor microbiomes, for example containing *Listeria monocytogenes, Shigella sonnei*, or *Salmonella enterica*, and to explore which secreted *L. reuteri* AMBV339 metabolites can inhibit the growth of these pathogens, potentially including lactic acid, reuterin and reutericyclin that have been described as antimicrobial metabolites of *L. reuteri* strains (69). Future clinical research with *L. reuteri* AMBV339 will explore its properties in the human gastrointestinal tract, as well as its capacity to enhance riboflavin levels and modulate the microbiome composition *in vivo*.

## Conclusion

We demonstrate that *L. reuteri* AMBV339 is a promising food biofortification candidate with a unique spontaneous riboflavin-overproducing phenotype. The capacity of *L. reuteri* AMBV339 for riboflavin production surpasses the non-genetically modified lactic acid bacteria described in literature, and can cover the daily riboflavin requirements in one glass of fermented plant-based or milk beverages. Furthermore, survival and uptake of riboflavin produced by *L. reuteri* AMBV339 in the simulated gastrointestinal dialysis model without perturbations of the healthy human microbiome highlight its immediate potential to provide riboflavin-related health benefits in humans and animals.

## Data Availability Statement

The datasets presented in this study can be found in online repositories. The names of the repository/repositories and accession number(s) are https://www.ebi.ac.uk/ena, PRJEB52196 and GCA_940926095 for the *L. reuteri* AMBV339 genome assembly.

## Ethics Statement

The studies involving human participants were reviewed and approved by the Committee of Medical Ethics UZA/UAntwerpen, Belgium. The patients/participants provided their written informed consent to participate in this study.

## Author Contributions

IS, SA, and SL conceptualized the research and wrote the original draft. IS, SA, AB, IE, AA, NH, and SL designed the experiments. SA, IS, AB, IE, SW, WVB, TE, AA, and NB conducted the experiments. IS, SA, AB, IE, SW, AA, and NB processed and analyzed the data. IS, SA, AB, IE, SW, PB, AA, SV, NH, and SL were involved in the interpretation of the results. IS, SA, AB, IE, and SW visualized the data. All authors reviewed, edited and approved the manuscript.

## Conflict of Interest

A patent application EP20210606.8 (owned by the University of Antwerp and with SA, IS, SW, and SL as inventors) has been submitted based on the results described in this manuscript. SL has received (research) funding from several companies, but they were not involved in this study. The remaining authors declare that the research was conducted in the absence of any commercial or financial relationships that could be construed as a potential conflict of interest.

## Publisher’s Note

All claims expressed in this article are solely those of the authors and do not necessarily represent those of their affiliated organizations, or those of the publisher, the editors and the reviewers. Any product that may be evaluated in this article, or claim that may be made by its manufacturer, is not guaranteed or endorsed by the publisher.

## Footnotes

1
https://github.com/tseemann/shovill

2
https://patricbrc.org/

3
https://www.genome.jp/kegg/pathway.html

4
https://www.fpbase.org

5
https://github.com/tseemann/abricate

6
github.com/Swittouck/tidyamplicons

7
github.com/Sarahtopia/ambv339_gidm

8
https://isala.be/en/
