# Supplementary material for: Spontaneous Riboflavin-Overproducing Limosilactobacillus reuteri for Biofortification of Fermented Foods
Source: Front Nutr. 2022 Jun 9;9:916607. doi: 10.3389/fnut.2022.916607 (PMC9218631; doi:10.3389/fnut.2022.916607)
Supplement: Supplementary file 1 [file Data_Sheet_1.PDF]

Supplementary Material

Supplementary table S1. Gas production (volume percent) and pressure in the headspace of *Limosilactobacillus reuteri* cultures grown in MRS broth.

	**Blank**				***L. reuteri* AMBV339**				***L. reuteri* AMBV336**				***L. reuteri* RC-14**			
**time (h)**	**O_2 (%)**	**N_2 (%)**	**CO_2 (%)**	**Pressure (bar)**	**O_2 (%)**	**N_2 (%)**	**CO_2 (%)**	**Pressure (bar)**	**O_2 (%)**	**N_2 (%)**	**CO_2 (%)**	**Pressure (bar)**	**O_2 (%)**	**N_2 (%)**	**CO_2 (%)**	**Pressure (bar)**
**0**	19	83	0	0	19	83	0	0	19	83	0	0	19	83	0	0
**2**	19	83	0		20	83	0		20	82	0		19	83	0	
**4**	19	83	0		19	82	1		19	82	1		19	83	0	
**6**	19	84	0	-0.15	11	46	48	0.85	10	46	49	0.7	17	72	16	0.2
**24**	17	84		-0.15	8	35	62	1.1	8	35	63	1.05	8	38	61	1.1

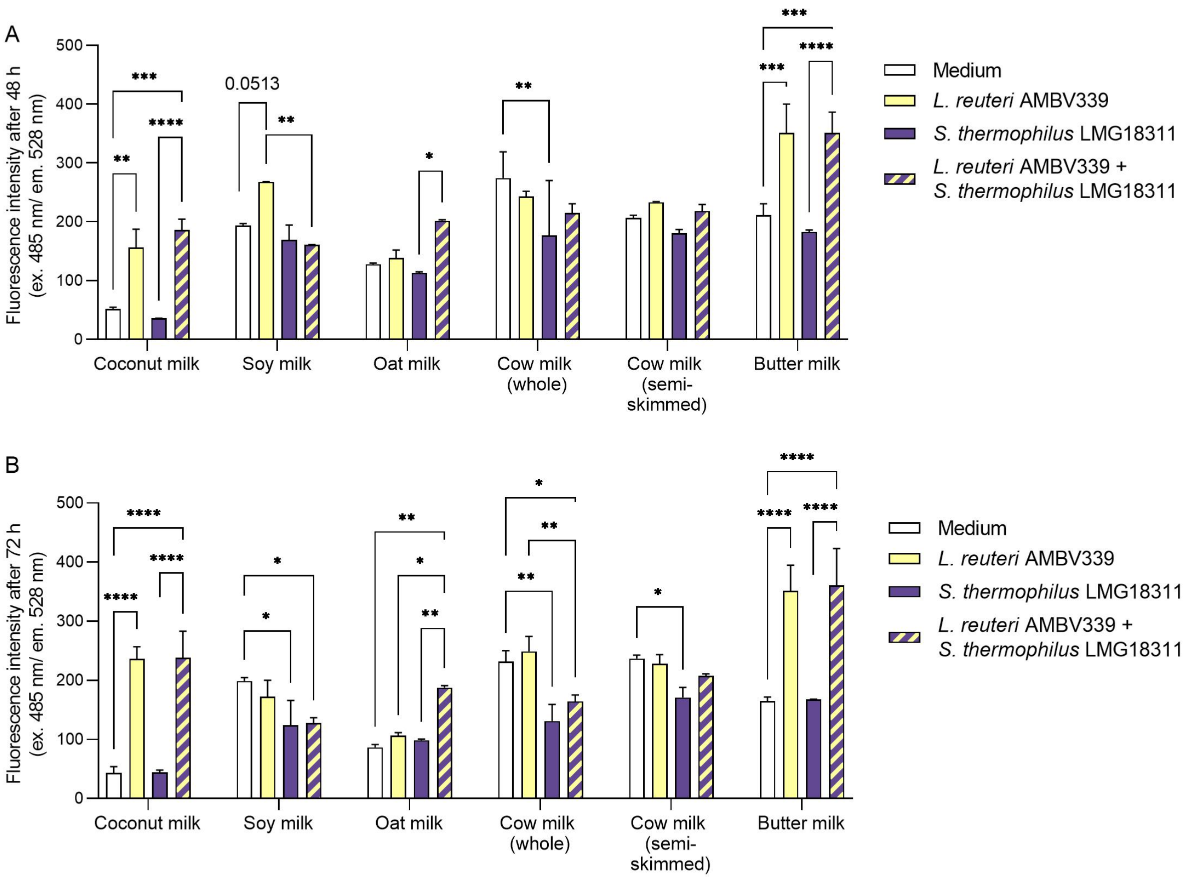

Supplementary figure S1. Riboflavin production in (A) 48 and (B) 72 h cultures of *L. reuteri* AMBV339 and *S. thermophilus* LMG18311 alone or combined in a coconut, soy or oat beverage, whole and semi-skimmed cow milk, or butter milk. Data is presented as means ± SD. *p<0.05, **p<0.01, ***p<0.001, ****p<0.0001 or depicted p value; comparisons were made with the medium condition, or with the *L. reuteri* AMBV339 + *S. thermophilus* condition.

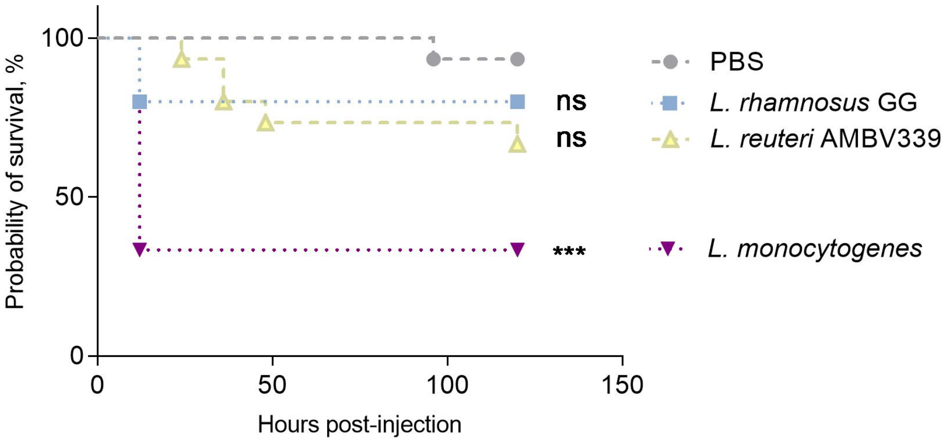

Supplementary figure S2. Survival of *G. mellonella* larvae injected with *L. reuteri* AMBV339, commercial probiotic *Lacticaseibacillus rhamnosus* GG or pathogen *Listeria monocytogenes* over the course of 144 hours post-injection. ns, non-significant, *** $p<0.001$, according to pairwise comparisons with the PBS condition. Fifteen larvae per group were used. All of the non-injected larvae survived during the timespan of the treatment.

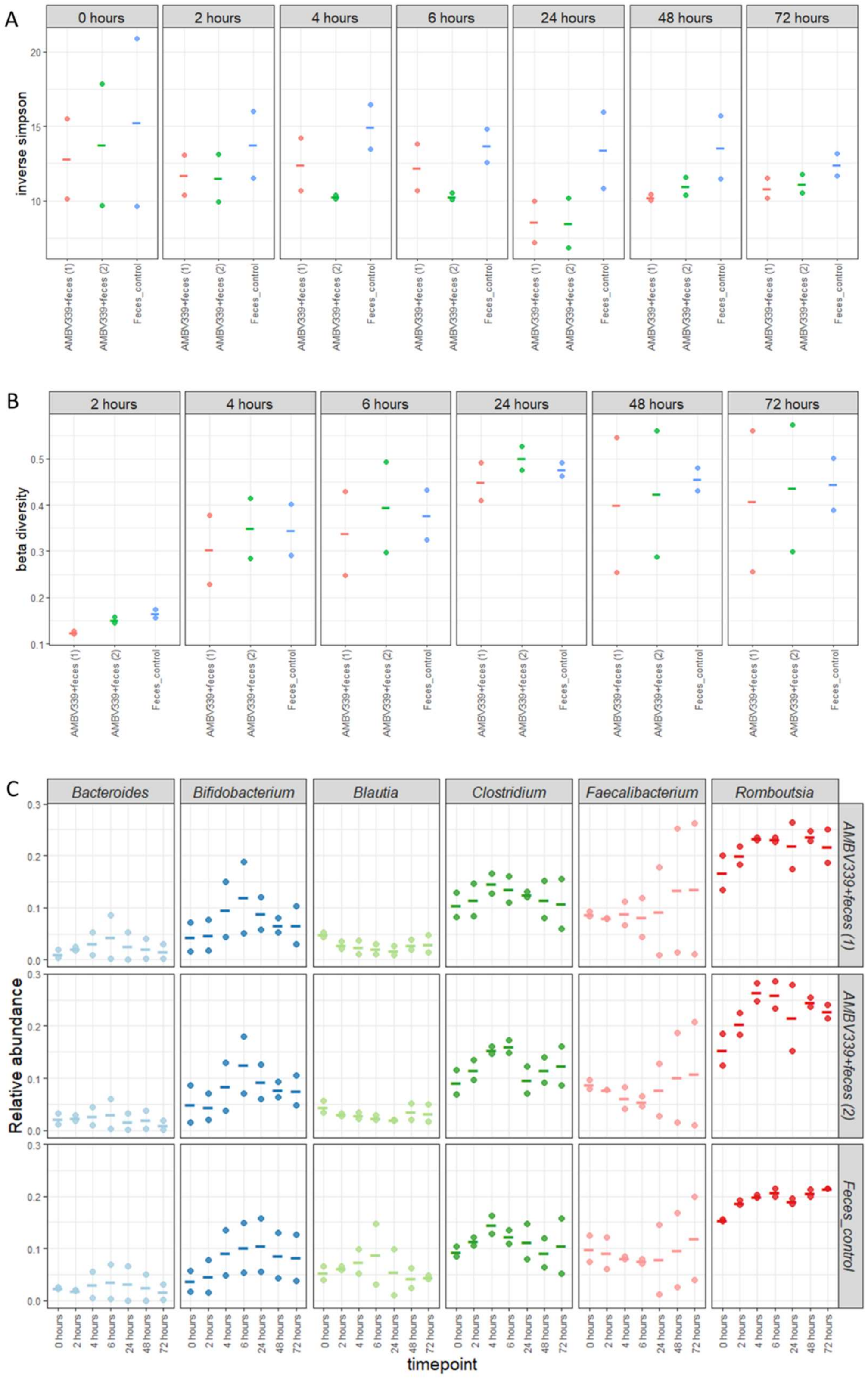
A
0 hours
2 hours
4 hours
6 hours
24 hours
48 hours
72 hours
inverse simpson
AMBV339+feces (1)
AMBV339+feces (2)
Feces_control
B
beta diversity
C
Bacteroides
Bifidobacterium
Blautia
Clostridium
Faecalibacterium
Romboutsia
AMBV339+feces (1)
AMBV339+feces (2)
Feces_control
Relative abundance
timepoint

Supplementary figure S3. (A) The Inverse Simpson microbiome diversity index over seven timepoints and (B) Bray-Curtis dissimilarities between the microbiome composition of the *L. reuteri* AMBV339 in feces condition (in duplicate) and feces as such over time compared to the start (0h). The line represents the mean. (C) Relative abundances of six dominant gut genera (i.e., *Bacteriodes, Bifidobacterium, Blautia, Clostridium, Faecalibacterium* and *Romboutsia*) determined at seven time points of the GIDM-colon for *L. reuteri* AMBV339 in feces condition (in duplicate) and feces as such. Each timepoint and condition contain samples from experiments with *L. reuteri* AMBV339 in saline (0.9% NaCl) and coconut beverage. *L. reuteri* AMBV339 reads were removed before microbiome analyses.

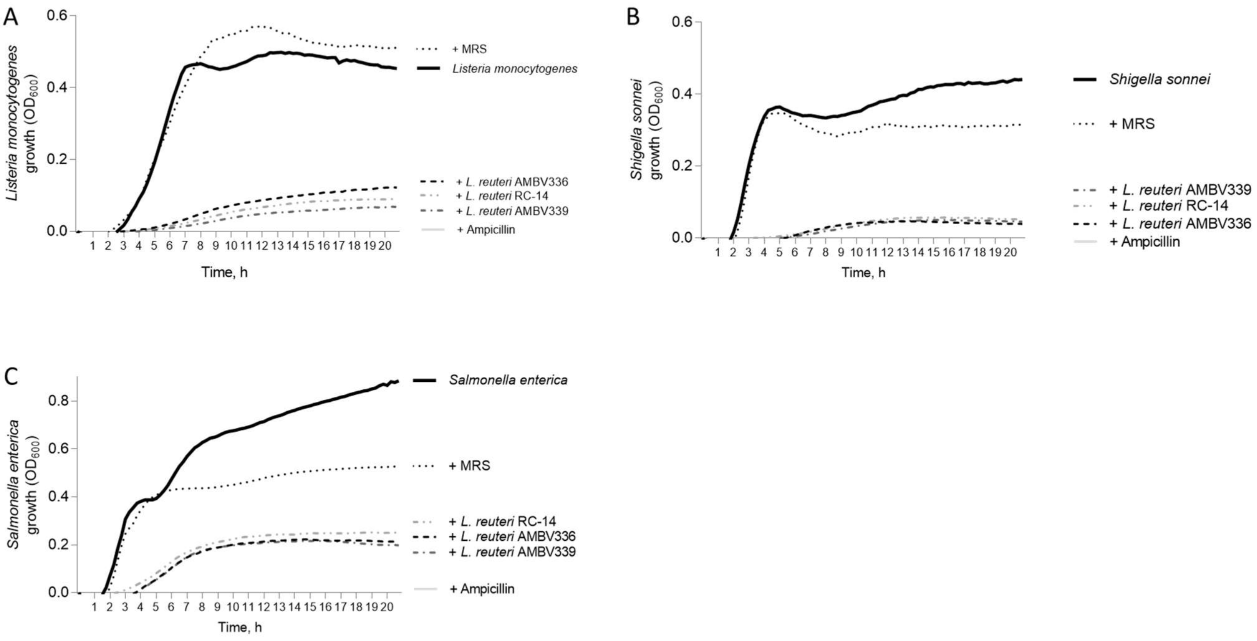

Supplementary figure S4. Longitudinal growth inhibition of enteric pathogens (A) *Listeria monocytogenes*, (B) *Shigella sonnei* and (C) *Salmonella enterica* under influence of supernatants of *L. reuteri* AMBV336, *L. reuteri* AMBV339 and *L. reuteri* RC-14 in MRS. Supernatants were added at 1:5 dilution and MRS medium was used as control. Data is presented as growth curve means per condition.
